# Improved Clinical Risk Stratification in Patients with Long QT Syndrome? Novel Insights from Multi-Channel ECGs

**DOI:** 10.1371/journal.pone.0158085

**Published:** 2016-07-05

**Authors:** Alexander Samol, Mehmet Gönes, Sven Zumhagen, Hans-Jürgen Bruns, Matthias Paul, Christian Vahlhaus, Johannes Waltenberger, Eric Schulze-Bahr, Lars Eckardt, Gerold Mönnig

**Affiliations:** 1 Division of Cardiology, Department of Cardiovascular Medicine, University Hospital Münster, Münster, Germany; 2 Division of Electrophysiology, Department of Cardiovascular Medicine, University Hospital Münster, Münster, Germany; 3 Institute for Genetics of Heart Diseases (IfGH), Department of Cardiovascular Medicine, University Hospital Münster, Münster, Germany; University of Tampere, FINLAND

## Abstract

**Background:**

We investigated whether multichannel ECG-recordings are useful to risk-stratify patients with congenital long-QT syndrome (LQTS) for risk of sudden cardiac death under optimized medical treatment.

**Methods:**

In 34 LQTS-patients (11 male; age 31±13 years, QTc 478±51ms; LQT1 n = 8, LQT2 n = 15) we performed a standard 12-channel ECG and a 120-channel body surface potential mapping. The occurrence of clinical events (CE; syncope, torsade de pointes (TdP), sudden cardiac arrest (SCA)) was documented and correlated with different ECG-parameters in all lead positions.

**Results:**

Seven patients developed TdP, four survived SCA and 12 experienced syncope. 12/34 had at least one CE. CE was associated with a longer QTc-interval (519±43ms vs. 458±42ms; p = 0.001), a lower T-wave integral (TWI) on the left upper chest (-1.2±74.4mV*ms vs. 63.0±29.7mV*ms; p = 0.001), a lower range of T-wave amplitude (TWA) in the region of chest lead V8 (0.10±0.08mV vs. 0.18±0.07mV; p = 0.008) and a longer T-peak-T-end time (TpTe) in lead V1 (98±23ms vs. 78±26ms; p = 0.04). Receiver-operating-characteristic (ROC) analyses revealed a sensitivity of 96% and a specificity of 75% (area under curve (AUC) 0.89±0.06, p = 0.001) at a cut-off value of 26.8mV*ms for prediction of CE by TWI, a sensitivity of 86% and a specificity of 83% at a cut-off value of 0.11mV (AUC 0.83±0.09, p = 0.002) for prediction of CE by TWA and a sensitivity of 83% and a specificity of 73% at a cut-off value of 87ms (AUC 0.80±0.07, p = 0.005) for prediction of CE by TpTe.

**Conclusions:**

Occurrence of CE in LQTS-patients seems to be associated with a prolonged, low-amplitude T-wave.

## Introduction

The congenital long QT syndrome (LQTS) is a channelopathy with actually 13 known different genotypes and more than 500 identified mutations affecting ventricular repolarization. Its penetrance is variable and clinical symptoms range from asymptomatic patients to significant cardiac events such as syncope, torsade de pointes (TdP) tachycardia, sudden cardiac arrest (SCA) or sudden cardiac death (SCD) [1]. Diagnostic criteria were developed including electrocardiographic parameters like a heart rate corrected QT prolongation above 450ms in male patients and 460ms in female patients, occurrence of TdP, detection of T-wave alternans, clinical features like syncope, congenital deafness and a positive family history [2, 3]. In addition to QT prolongation, patients with LQTS often show more or less specific variations of the T-wave [4]. Besides lifestyle modification, i. e. avoidance of QT prolonging drugs, potassium or magnesium deficiency, and trigger situations (sudden noises that lead to arousal in LQT2, competitive sports or other sympathetic activation effecting situations in LQT1) [5, 6] beta-blocker therapy was found to reduce occurrence of SCA/SCD in patients with LQTS significantly [7]. On the other hand it was shown that patients with syncope under beta-blocker therapy had a relative high risk for developing SCD [7]. Several other risk factors in patients with LQTS for the occurrence of life-threatening events have been identified: The risk for developing cardiac events is greater in patients with QTc interval > 500ms [5]. Hobbs et al. showed in a large study with more than 2,700 patients that a QTc interval > 530ms was associated with a more than twice higher risk for developing cardiac events [8]. Other risk factors for cardiac events are gender and age [7], occurrence of previous symptoms [8, 9] and a family history of SCD [10]. Additional electrocardiographic parameters from standard electrocardiography (ECG) like T_peak_-T_end_/QT-ratio promises additional benefit in risk stratification of patients with (congenital) arrhythmias [11].

Body surface mapping (BSM) is a dependable tool for the detection of heterogeneities of ventricular repolarization unrevealed by conventional ECG recording [12]. Up to now, only two reports have been published on repolarization abnormalities that were identified by body surface QRST-integral- and ST-T-integral mapping in a small group of non-genotyped LQTS patients [13] and very recently, by electrocardiographic imaging (ECGI) in a group of 25 genotyped LQTS patients [14]. The usefulness of BSM in risk stratification of LQT patients has not been shown so far. Therefore, we retrospectively analyzed different ECG-parameters from 12-channel and from multichannel ECG-recordings and correlated these with the occurrence of clinical events.

## Materials and Methods

### Study patients

#### Patients with Long QT syndrome

This study was performed according the Declarations of Helsinki and was approved by the Ethical Board of the Ärztekammer Westfalen Münster as part of the SFB 656, project C1. Thirty-four consecutive patients with non-acquired LQTS (11 male, mean age 31±13 years) were enrolled in this study after written informed consent was obtained. All patients underwent detailed non-invasive diagnostic procedures including 12-channel ECG, treadmill test and transthoracic echocardiography. In addition, genetic analyses were performed in 29 patients, five patients declined DNA analyses. Medical history was documented with respect to syncope, TdP-tachycardia and SCA. All patients were under beta-blocker therapy (Metoprolol n = 20; Bisoprolol n = 13; Propranolol n = 1). LQTS patients were regularly followed in our outpatient clinic. Follow-up period started with the beginning of beta-blocker therapy, BSM recordings were performed on a following outpatient clinic visit. 10 patients were fitted with an implanted cardioverter defibrillator (ICD) before inclusion in our study because of documented (n = 4) or assumed (n = 6) arrhythmias, ICD interrogations were scheduled every 3 months. Therapies were classified as appropriate if the stored electrograms and/or RR intervals confirmed that the tachyarrhythmia was sustained and fulfilled the device criteria for ventricular origin before the first ICD treatment. To exclude a potential confounding effect of cardiac memory caused by temporary pacing only patients without pacemaker-dependence were included in our study (i. e. an overall percentage of zero in the month before BSM recording).

#### Genetic analysis

Genomic deoxyribonucleic acid (DNA) was extracted from peripheral blood leucocytes following standard procedures as reported before [15, 16]. LQTS specific genes were analyzed by direct sequencing. All coding gene regions of the probands DNA were analyzed by polymerase chain reaction and followed by direct sequencing on a solid support. Analyses were performed following pre-defined analysis levels and were stopped after a level was completed or a mutation was found [15]. In members of families with known mutations (n = 16), genetic analyses were only performed in the mutant gene region. Each patient gave written informed consent before genetic analysis; the study was in accordance with the latest revised version of the Declaration of Helsinki and with recommendations of the local ethics committee.

#### Control group

Thirteen healthy volunteers (mean age 54±15 years) with no history, signs, or symptoms of coronary artery disease or underlying cardiomyopathy served as a control group.

### Data acquisition and data analysis

#### Body surface mapping

**Fig 1:**
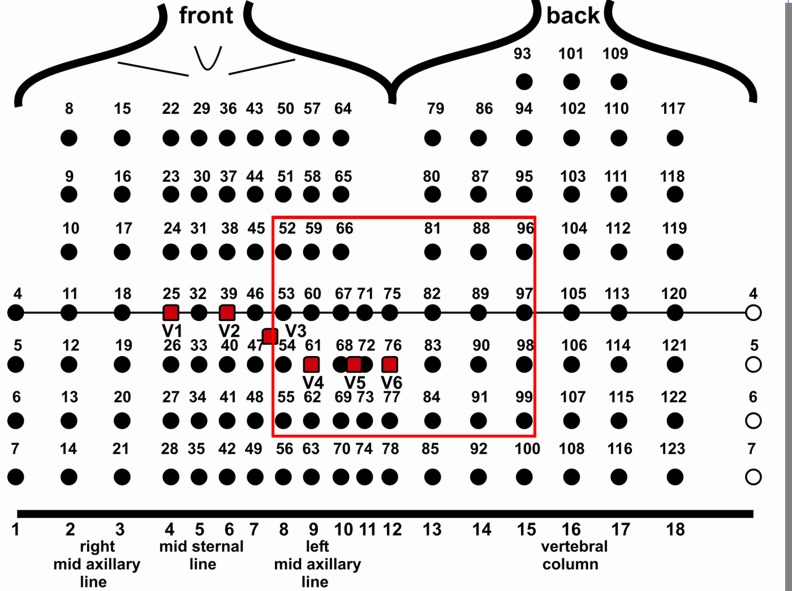
Array of 120 electrodes for the recording of unipolar body surface potentials. Red rectangles indicate the positions of standard chest leads V1 to V6. Red frame indicates the region of interest of Fig 3.

BSM measurements were performed under beta-blocker medication but in the absence of any concomitant antiarrhythmic, QT prolonging or sedative medication. In LQTS-patients with an ICD, only patients with a pacemaker-stimulation percentage of zero in the month before BSM recording were included. As reported before, BSM electrodes were applied to patient´s chest in vertical strips (Fig 1) [17]. All electrodes (Foxmed GmbH, Idstein, Germany) were equipped with a 10 mm diameter Ag/AgCl sensor embedded in epoxy housing with a 2 mm gel cavity. Inter-electrode distance on the strips was 50 mm in vertical direction and minimal 30 mm in horizontal direction (Fig 1). The recording of the ECG signals of the 120 unipolar leads referred to Wilson central terminal was performed simultaneously. The recorded signals were amplified, bandpass-filtered with a spectrum between 0.16 and 400 Hz and A/D converted to 16 bit samples (0.5 μV least significant bit) using a sampling rate of 1 kHz (Mark-6, Biosemi, Amsterdam, Netherlands). Excessively noisy or artifacts containing leads were excluded from further analysis. All recordings were performed for a period of five min. after a resting period of 5 min. in supine position under stable sinus rhythm. As reported before, recorded signals without any offset variation due to respiratory motion and with high signal-to-noise ratio were selected for off-line single-beat analysis [17]. T-wave integral, QRST integral and T-wave amplitude range (i. e. the absolute value of maximum positive and negative T-wave peak) in each channel including standard chest leads V_1_-V_6_ was measured in all patients with LQTS and compared between patients with occurred clinical events to those LQTS patients without clinical events and controls. The on- and the offset of QRS-complex and the T-wave were defined manually and marked by digital calipers independently by three experienced cardiologists (A.S., G.M, and M.P.) according to peak-slope-intercept technique, using the PQ-interval as reference zero.

#### 12-lead surface ECG

A 12-lead surface ECG recorded with a paper speed of 50mm/s under beta-blocker therapy in the absence of any concomitant anti-arrhythmic medication or QT prolonging drugs at the time of the BSM recording was available in all patients and controls. Blinded for clinical details, these ECGs were quantitatively analyzed using digital measure software (DatInf® Measure, Germany). In all 12 standard leads the following parameters were determined: RR-distance, R-wave amplitude, T-wave amplitude, PQ interval, QRS duration, QT interval, T_peak_-T_end_ time, T_peak_-T_end_/QT-ratio.

#### Statistical analysis

Statistical analyses were performed using IBM SPSS Statistics (version 22 for MAC, IBM Corporation, Somers, NY, USA). In case of metric target variables non-parametric analysis of variance (ANOVA) with post-hoc testing adjusted for multiplicity applying the closed test principle was used. Differences of binary target variables were assessed by using the χ^2^ test. P values ≤ .05 were regarded significant. Receiver-operating-characteristic (ROC) analyses were performed of the T-wave integral and the T-wave amplitude range for each BSM lead and of T_peak_-T_end_-interval for each lead of 12-channel ECG. Areas under curve (AUC) are presented as mean ± standard error of the mean (SEM); all other data are expressed as mean ± standard deviation (SD).

## Results

### Clinical characteristics

Demographic characteristics of the study patients are depicted in Table 1. Twelve patients with LQTS developed at least one clinical event (CE; i. e. syncope n = 12, TdP n = 7 or SCA n = 4) under beta-blocker therapy during a mean Follow-up time of 19±18 months. There were no differences as to the age or body mass index at BSM recording between the patients with CE and without CE (Table 1). Ten patients with LQTS (29%) had received an automatic cardioverter-defibrillator (ICD) before the beginning of follow up, all SCA episodes occurred in ICD patients and were adequately treated by the device. Genetic analyses identified a LQT1 mutation in 8 patients (24%), LQT2 mutation in 15 patients (44%), LQT1 and LQT2 (double mutation) in one patient (3%), and LQT8 in one patient (3%). If no LQT1, LQT2 or LQT3 mutations were identified (n = 4) or patients declined genetic analysis (n = 5) these patients were declared as LQTx (n = 9). One patient that declined the genetic test presented typical ECG findings compatible to LQTS and his family member was identified as LQT1. There was another that declined the genetic test who had a daughter genetically diagnosed as LQT3 and a history of recurrent syncope. 16 patients had known mutations in their family, 18 were propositus. Results of genetic analyses are shown in Table 2.

**Table 1:** Clinical and electrocardiographic characteristics of study patients. CE clinical event; F female; ICD implantable cardioverter defibrillator; LQTS long-QT syndrome; M male; n number; ns not significant; TWAR T-wave amplitude range; TWI T-wave integral. Values are expressed as mean ± SD where applicable.

	LQTS			P value*	Controls	P value#
	All	CE +	CE -			
**Clinical characteristics**						
Patients, n	34	12	22		13	
Age, years	31±13	28±13	33±12	ns	54±15	0.01
Sex (M/F)	12 / 22^*^	3 / 9^‡^	9 / 13^‡^	ns	6 / 7^†^	ns
Body mass index	26.6 ± 3.2	27.5 ± 2.0	25.3 ± 3.0	ns	26.3 ± 3.1	ns
ICD, n (%)	10 (29)^*^	6 (55)^†^	4 (17)^†^	0.06	0 (0)	<0.001
**ECG characteristics**						
QTc duration (ms)	478±51	519±43	458±42	0.001	429±25	0.03
TWI channel 60 (mV*ms)	40.3±58.1	-1.2±74.4	63.0±29.7	0.001	41.6±33.3	ns
TWAR channel 90 (mV)	0.15±0.08	0.10±0.08	0.18±0.07	0.008	0.14±0.06	ns
T_peak_-T_end_-interval V_1_ (ms)	85.4±26.7	98.3±23.4	78.4±26.32	0.04	86.0±12.3	ns
T_peak_-T_end_/QTc V_1_	0.184±0.049	0.198±0.048	0.176±0.048	ns	0.216±0.035	0.04
heartrate (bpm)	59±12	56±13	61±12	ns	66±11	ns
QT duration (ms)	488±64	549±50	457±47	0.001	408±39	0.003
QRS duration (ms)	84±16	86±15	83±17	ns	89±07	ns
RR interval (ms)	985±205	998±268	977±168	ns	883±125	ns

# = p LQTS vs. control

* = p CE+ vs. CE -.

**Table 2:** Results of genetic analysis, beta-blocker dosage and clinical events of our study patients; # = patients with clinical event.

Patient	remarks	gene	nucleic acid	protein	Beta-blocker
**1 #**	no mutations found in standard gene				Bisoprolol 5mg/d
**2**	no mutations found in standard gene				Metoprolol 200 mg/d
**3**	no mutations found in standard gene				Metoprolol 150mg/d
**4 #**		LQT2	G1714T	G572C	Propranolol 180mg/d
**5**		LQT2	G1714T	G572C	Metoprolol 100mg/d
**6 #**		LQT2	G1714T	G572C	Metoprolol 150mg/d
**7**		LQT2	2162 C>G	P721R	Metoprolol 100 mg/d
**8**		LQT2	2162 C>G	P721R	Bisoprolol 10mg/d
**9**		LQT2	2162 C>G	P721R	Bisoprolol 10mg/d
**10**		LQT2	2616 delC	G873fsX877	Metoprolol 100mg/d
**11**		LQT1	G355C	Gly119Arg	Metoprolol 200mg/d
**12**	no genetic analysis				Metoprolol 150mg/d
**13**	no mutations found in standard gene				Metoprolol 200mg/d
**14 #**		LQT2	C1283T	S428L	Metoprolol 200mg/d
**15 #**	no genetic analysis, LQT 1 mutation in family				Bisoprolol 10mg/d
**16**		LQT1	1015-1017delTTC	Phe339 del	Bisoprolol 5mg/d
**17**	no genetic analysis, daughter with LQT 3			-	Bisoprolol 10mg/d
**18**		LQT2	G1714T	G572C	Bisolprolol 5mg/d
**19**		LQT1	C935T	T312I	Bisoprolol 5mg/d
**20**		LQT1	T752C	Leu251Pro	Metoprolol 100mg/d
**21**		LQT8	5399 C>T	T1752I	Bisoprolol 10mg/d
**22**		LQT1	T839C	Val280Ala	Metoprolol 100mg/d
**23 #**	no genetic analysis				Metoprolol 100mg/d
**24**		LQT1	T910C	Trp304Arg	Metoprolol 200mg/d
**25 #**		LQT2	2966–3 c>g	A990WfsX5	Bisoprolol 5mg/d
**26**		LQT2	1870insC	S624fsX654	Metoprolol 100mg/d
**27 #**		LQT1+LQT2	LQT1: 1892-1911del / LQT2: G211A	LQT1: P631HfsX14 / LQT2: G71R	Metoprolol 200mg/d
**28**		LQT2	2062 C>T	Q688X	Bisoprolol 10mg/d
**29**		LQT2	2775 insG	P926Afsx14	Bisoprolol 5mg/d
**30**	no genetic analysis				Bisprolol 5mg/d
**31 #**		LQT2	G1681C	A561P	Metoprolol 150mg/d
**32**		LQT2	2062 C>T	Q688X	Metoprolol 200mg/d
**33 #**		LQT1	1066 C>T	Gln356*	Metoprolol 150mg/d
**34 #**		LQT1	940 G>A	Gly314Ser	Metoprolol 100mg/d

### 12-Lead standard surface ECG in the study population

**Fig 2:**
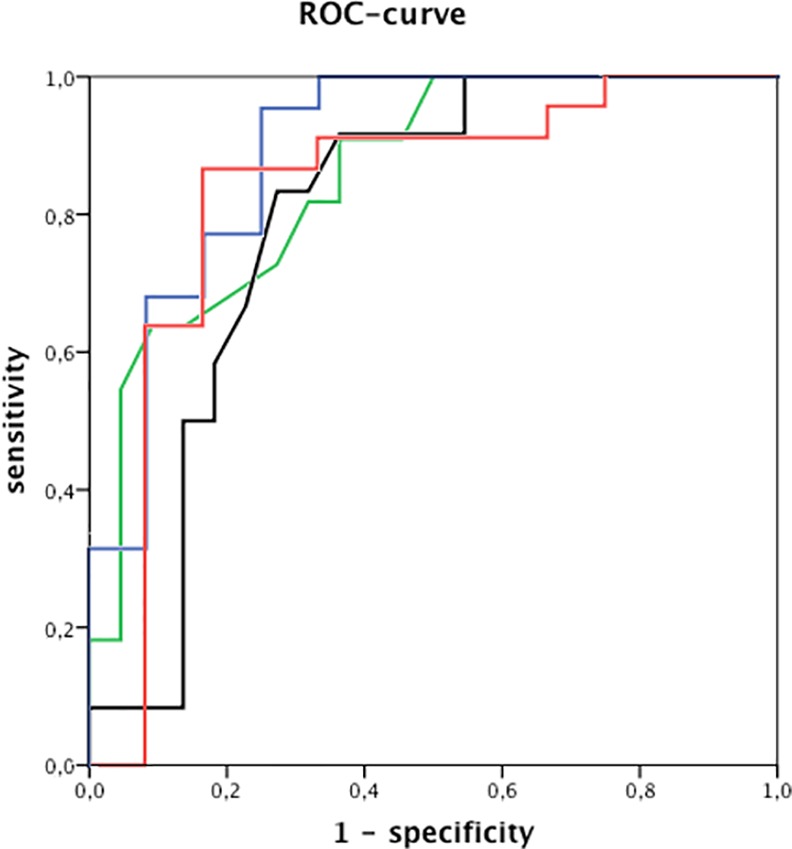
Receiver operating characteristic curve analysis with T-wave integral of the key channel 60 (blue lines; AUC 0.89), T-wave amplitude range of the key channel 90 (red lines; AUC 0.83), T_peak_-T_end_-interval in standard chest lead V_1_ (black lines; AUC 0.8) and QTc time from standard ECG (green line; AUC 0.86).

BSM and conventional 12-lead surface ECGs were recorded during sinus rhythm in all patients and controls. Patients with LQTS had significantly longer QTc duration than controls (476±52ms vs. 429±25ms; p = 0.03). Patients that developed CE had a significantly longer QTc interval than patients without CE (519±43ms vs. 458±42ms; p = 0.001, Fig 2).

ROC analysis to predict occurrence of CE with QTc duration revealed at a cut-off value of 488ms a sensitivity of 82% and a specificity of 68% (AUC 0.86±0.07, p = 0.001).

Analysis of alternative ECG parameters obtained from 12-lead standard ECG identified a significantly longer T_peak_-T_end_ time in lead V_1_ in patients with LQTS and a CE than in patients without a CE (98±23ms vs. 78±26ms; p = 0.04). ROC analysis for prediction of CE by T_peak_-T_end_ time revealed a sensitivity of 83% and a specificity of 73% at a cut-off value of 87ms (AUC 0.8±0.07, p = 0.005, Fig 2)

However, T_peak_-T_end_ time was not significantly different in LQTS patients when compared to controls. In addition, there was no detectable increase in the difference in this alternative ECG parameter compared to controls in patients with more than one type of CE (syncope±TdP±SCA).

### BSM in study population

**Fig 3:**
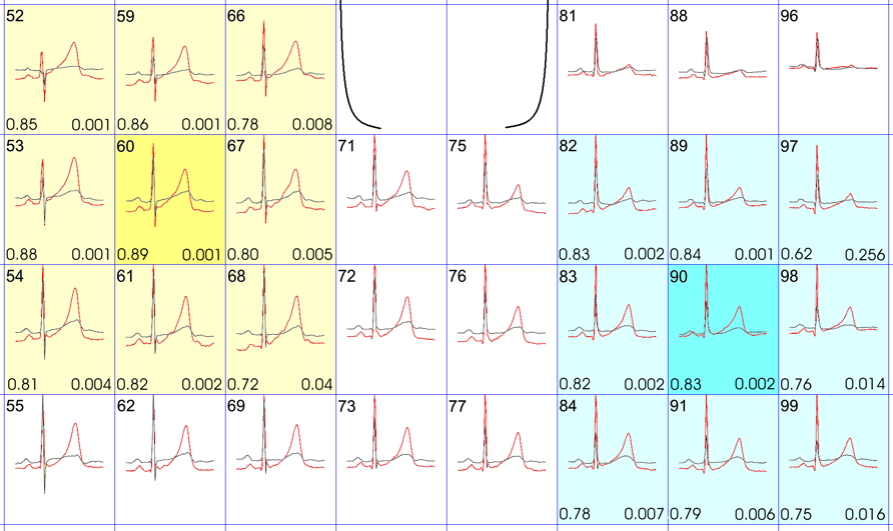
Superimposition of two typical BSM recordings from the region of interest: Red curves from a patient with LQT2 and without clinical event, black curves from a patient with LQT2 and with clinical event. Region of interest for T-wave integral is yellow highlighted, region of interest for T-wave amplitude range is blue highlighted. Channel numbers are listed on the left top of each rectangle, Area under curve values from ROC analysis are listed on the left bottom and P values of ROC analysis are listed on the right bottom of each rectangle

T-wave integral, QRST integral and T-wave amplitude were calculated for all BSM channels. Occurrence of CE was associated with a significantly lower T-wave integral (TWI) in an alternative lead position localized on the left upper chest approximately one intercostal space above standard chest lead V_4_ (-2.6±77.9mV*ms vs. 60.0±30.4mV*ms; p = 0.002, Fig 3). At a cut-off value of 26.8mV*ms ROC analysis for prediction of CE by TWI revealed a sensitivity of 96% and a specificity of 75% (AUC 0.89±0.06, p<0.001, Fig 2). These findings were also reproducible in adjacent channels and uncoupled slowly with increasing distance (Fig 3). In other torso regions no statistical significance was observed to predict events. However, TWI was not significantly different between the whole patient group with LQTS independently of CE and controls.

Occurrence of CE was also associated with a significantly lower QRST integral (QRSTI) in the same region as TWI (9.9±93.8mV*ms vs. 90.2±35.0mV*ms; p = 0.001). Nevertheless, ROC analysis showed no diagnostic benefit compared to TWI. At a cut-off value of 60.5mV*ms a sensitivity of 77% and a specificity of 83% were obtained (AUC 0.86±0.07, p = 0.001).

Occurrence of CE was also associated with a significantly lower range of T-wave amplitude in an alternative lead position in the region of chest lead V8 (0.11±0.08mV vs. 0.19±0.1mV; p = 0.02, Fig 3). ROC analysis for prediction of CE by T-wave amplitude range revealed a sensitivity of 86% and a specificity of 83% at a cut-off value of 0.11mV (AUC 0.83±0.09, p = 0.002, Fig 2). These findings were also reproducible in adjacent channels and uncoupled slowly with increasing distance (Fig 3). In other torso regions no statistical significance was observed to predict events. Again, we were not able to show significantly differences in T-wave amplitude range between the whole LQTS patient group and controls.

### Usefulness of the alternative parameters in LQT2 subgroup

To exclude potential bias of our results caused by the inhomogeneity of the subgroups, we performed ROC analysis of T-wave integral, QRST integral, T-wave amplitude range and T_peak_-T_end_-interval in LQT2 patients representing the largest subgroup of our cohort. In LQT2 patients (n = 16) occurrence of CE (n = 6) was also associated with a lower TWI (7.8±15.4 vs. 43.3±23.4 mV*ms, p = 0.007), a significantly lower QRSTI (29.5±23.8 vs. 83.3±34.6 mV*ms, p = 0.006) and a significantly lower T-wave amplitude in this particular area (0.08±0.04 vs. 0.2±0.14 mV, p = 0.03). In this subgroup the most significant channel for prediction of CE by T-wave amplitude range was number 98, which is the adjacent channel to number 90 and in the region of standard chest lead V8. Nevertheless in this subgroup T_peak_-T_end_ time was not significantly longer in LQTS patients with CE compared to LQTS patients without CE.

## Discussion

Our main intention was to identify additional ECG parameters for risk stratification and to find a potential way in eliminating differences in T-wave morphology between different subgroups by using T-wave parameters that describe T-wave characteristics independent of their morphology: Therefore, we used T-wave integral (TWI) and T-wave amplitude range. Indeed these two parameters varied not significantly between LQT1 and LQT2 patients in our cohort. In contrast, T-peak-Tend time failed to show significant differences in the LQT2 subgroup between low and high-risk patients.

In addition to QT prolongation more or less specific T-wave morphologies have been described for LQTS subgroups: Moss et al. reported a broad based T-wave pattern in patients with LQT1, a low amplitude T-wave with bifid T-waves in LQT2 and a late onset narrow peaked T-wave in LQT3 patients [4]. Nevertheless, other authors described broad variations in morphology of T-wave in specific subgroups [18], a fact that we also found in our collective. In our two largest subgroups LQT1 and LQT2 we found a trend towards the described typical T-wave patterns, but also totally differences in morphology even between family members carrying identical mutations.

The eponymous QT prolongation has been investigated in several studies for diagnosis and for risk stratification [19]. In general, heart-rate corrected QT intervals >450ms in male and >460 ms in female are regarded suggestive for LQTS. The standard ECG leads II or V5 as second choice seems to be most appropriate for QT duration measurement [15]. It is also one of the most important risk factors for SCD in LQTS patients: Schwartz et al. showed that patients with a QTc prolongation > 500ms had a significant higher risk for cardiac events compared to patients with QTc durations < 500ms [5]. In a large cohort of more than 2700 participants from the Long QT Syndrome Registry it was shown, that in patients with a QTc prolongation > 530ms that risk for cardiac events was more than twice higher compared to patients with QT prolongations < 530ms. We could demonstrate an exponential increasing risk for cardiac events in LQTS patients between deciles with increasing QT durations[15]. In our study cohort, patients with CE under beta blocker treatment showed significant higher event rates in patients with longer QTc. Mean QTc in patients with CE was 61ms longer compared to LQTS patients without CE and ROC analysis revealed a moderate sensitivity and specifity at a cut-off value of 488ms, a value close to the standard 500ms.

Many other non-electrocardiographic risk factors have been identified in patients with LQTS like gender and age, which are partially specific for distinct LQTS subtypes [7]. Because of the small size and the even smaller size of the LQTS subgroups in our cohort, we were not able to perform comparing analysis of these parameters in our study.

Analyses of alternative ECG parameters like T_peak_-T_end_-interval have been successfully used for risk stratification in other electrical heart diseases and in LQTS [11]. In LQTS patients Lubinski et al. reported a prolonged T_peak_-T_end_-ratio in LQTS patients compared to controls especially during night sleep hours [20]. Another study described exercise-induced differences in T_peak_-T_end_-interval accentuation in LQT1 but not in LQT2 patients combined with an increased propensity to develop exercise induced TdP in LQT1 patients [21]. In patients with acquired LQTS T-peak-T-end/QT ratio was identified as a better predictor of TdP as compared to QTc interval or QT dispersion [22]. In our cohort T-peak-T-end time was a better predictor of cardiac events compared to QTc interval alone, the calculation of the ratio of T-peak-Tend-time and QTc interval showed no benefit in prediction of CE.

T-wave integral, a parameter also reflecting local repolarization abnormalities, showed a correlation with ventricular tachycardia inducibility during programmed ventricular stimulation in patients with arrhythmogenic right ventricular cardiomyopathy [17]. So far, one study investigated the usefulness of BSM in non-genotyped LQTS patients: De Ambroggio et al. reported an area of more negative QRST and ST-T intervals on the right anterior and inferior thorax in patients with idiopathic LQTS as compared to healthy controls [13]. They found no correlations between syncopal events and the described abnormalities in their study [13]. They assumed a reduced right sympathetic activity as potential reason for the electronegative area reflecting delayed ventricular repolarization in this local region [13]. In our cohort we found no diagnostic benefit in using QRST integral instead T-wave integral for diagnosis or risk stratification in LQTS patients in any region of the patient torso and the most significant differences were localized in the left anterior upper torso, as a potential marker for disturbances of sympathetic innervation in the left ventricle. In contrast to the work of de Ambroggio and co-worker our results are in accordance to the results of a previous study in our LQT patients of Kies et al. found a reduced ^123^I-MIBG tracer uptake in patients with LQTS in the anteroseptal segments of the left ventricle as a marker for impaired cardiac sympathetic function in this area [23]. This might explain our T-wave findings in the left precordial area. The described innervation defects were independent of the underlying genotype and clinical disease expression [23] which could also explain the independence of the underlying subtypes in T-wave integral differences in this distinct area between high and low risk patients in our present cohort.

Very recently, Vijayakumar et al. described steep repolarization discrepancies caused by local action potential duration prolongation in a small group of patients with congenital LQTS using ECGI [14]. This describes a potential substrate for reentrant arrhythmias and thus, steeper repolarization discrepancies may result in a higher risk for arrhythmias [14]. They assumed that these mechanisms are not detectable by surface ECG. On the other hand they described predominantly negative T-wave in regions with prolonged action potential durations compared to regions with normal action potential duration [14]. Therefore we used T-wave integral und T-amplitude range for risk stratification in optimal treated patients and indeed, we found significantly lower values in symptomatic patients.

## Study Limitations

This study included a relatively small number of patients. The patients are yet well characterized and underwent detailed diagnostic evaluation. However, due to the sample size, a potentially age- or LQT-subtype dependent cut-off level of T-wave integral, T-wave amplitude and T_peak_-T_end_ time remains to be elucidated. The healthy subjects we used as controls in our study were significantly older than our LQT-patients, age dependent differences may have influenced our results. The proposed alternative parameters and their cut-off values warrant prospective validation in a larger group of mere LQTS index patients. In addition, gender- and age-specific differences in patients with LQTS could not be addressed in this study.

## Conclusions

Analysis of T-wave integral, T-wave amplitude range and T-peak-T-end time discriminates LQTS patients with CE from those LQTS patients without CE. BSM measurements detected torso areas outside the standard lead positions with significantly altered T-wave integral and T-wave amplitude in particular in the left half of the torso. We were able to show a potential clinical value of our alternative parameters in identifying those patients with LQTS in whom CE occur despite of medical treatment and thus, potentially high risk patients. These findings have to be confirmed in large multi-center trials.

## Abbreviations

ANOVA: Analysis of variance
AUC: Area under curve
BSM: Body surface mapping
CE: Clinical event
DNA: Deoxyribonucleid acid
ECG: Electrocardiography
ECGI: Electrocardiographic imaging
ICD: Implanted cardioverter defibrillator
LQTS: Long-QT syndrome
QRSTI: QRST integral
ROC: Receiver operating characteristic
SCA: Sudden cardiac arrest
SCD: Sudden cardiac death
TdP: Torsade de pointes
TWI: T wave integral
